# Behavioral, contextual and biological factors associated with obesity during adolescence: A systematic review

**DOI:** 10.1371/journal.pone.0214941

**Published:** 2019-04-08

**Authors:** Janine Narciso, António José Silva, Vitor Rodrigues, Maria João Monteiro, António Almeida, Raquel Saavedra, Aldo Matos Costa

**Affiliations:** 1 Department of Sports Sciences, University of Beira Interior, Covilhã, Portugal; 2 Department of Sports, Exercise and Health Sciences, University of Trás-os-Montes e Alto Douro, Vila Real, Portugal; 3 Research Center in Sports Sciences, Health Sciences and Human Development, CIDESD, Vila Real, Portugal; 4 Superior School of Health, University of Trás-os-Montes e Alto Douro, Vila Real, Portugal; 5 Center for Health Technology and Services Research, CINTESIS, Porto, Portugal; 6 Health Sciences Research Center, CICS-UBI, Covilhã, Portugal; Centro Interdisciplinar de Performance Humana (CIPER), PORTUGAL

## Abstract

**Introduction/Objective:**

Adolescence is a critical period for the development of obesity. Obesity arises from a complex interaction between several factors, which are not yet fully understood. Therefore, the purpose of this review was to identify and assess the peer-reviewed scientific literature on the behavioral, contextual and biological factors associated with obesity in adolescents.

**Methods:**

PubMed and Scopus were systematically searched to identify prospective cohort studies concerning the relation between behavioral, contextual and biological factors and obesity in adolescents aged 10 to 18 years.

**Results:**

40 studies published between the year 2000 and 2018 were included. A positive consistent association between genetic factors and obesity during adolescence was found. Also, there is evidence to support the association between socioeconomic status and obesity. There was conflicting evidence for the contribution of dietary intake, physical activity, sedentary behavior, sleep, food store environment, school food environment. For the remaining factors no associations were found, or no conclusions could be drawn due to the limited number of studies identified.

**Conclusions:**

Further prospective studies that assess multiple obesity determinants simultaneously and use state-of-art measures are warranted to aid in the development of effective strategies and interventions to prevent obesity during adolescence.

## Introduction

Childhood obesity has been recognized as one of the major public health challenges of the 21^st^ century.[1] The number of obese children and adolescents aged 5 to 19 years increased from 11 million in 1975 to 124 million in 2016 and, an additional 213 million were overweight in 2016.[2] Two recent studies show that the rise in obesity in children and adolescents appears to be levelling off in high-income countries—albeit at high prevalence levels—but persists in some low and middle-income countries.[2,3]

Children and adolescents are exhibiting obesity-related health conditions such as type 2 diabetes, insulin resistance, hypertension, dyslipidemia, obstructive sleep apnea and fatty liver disease, which were previously only seen in adults.[4,5] In addition, obesity diminishes adolescents’ quality of life[6] and obese or overweight adolescents have a higher risk of becoming obese and/or centrally obese in adulthood.[7]

Adolescence is one of three critical periods for the development of obesity.[8] This might be explained by the fact that adolescence is a period characterized by changes in the quantity and location of body fat, a decline in physical activity and diet quality and an increase in sedentary behaviors. Additionally, from a psychosocial perspective, it is considered a period of increased risk of depression and anxiety.[9] Therefore, primary prevention of obesity in adolescents should be a public health priority.

Obesity results from a long-term energy imbalance between nutritional intake and activity, with the last being affected by physical activity (PA) and sedentary behavior.[10,11] Hence, it is no surprise that these behavioral factors have been the major focus of childhood obesity prevention and treatment interventions.[12] However, obesity arises from a complex interaction between genetic, epigenetic, environmental and behavioral factors, which are not yet fully understood.[13–15]

Due to the complexity of obesity, social ecological models have been proposed for its understanding, as they are useful in examining a wide range of factors that contribute to a complex health issue.[16] The conceptual framework adopted in this review was guided by both Faith and Kral conceptual model[17] and the Identifying Determinants of Eating and Activity study conceptual model for the etiology of childhood obesity.[16] Faith and Kral model[17] hypothesizes that genetic and social-environmental factors lead to the development of obesity through their independent influences on intermediary behavioral phenotypes (food intake and PA). These intermediary behavioral variables possibly will induce a positive energy balance that, if sustained, will promote obesity. The Identifying Determinants of Eating and Activity study conceptual model posits that obesity risk is most closely and directly affected by diet, activity-related and biological factors. It also posits that diet, activity and sedentary patterns impact both, biological factors and energy balance and, at the same time diet and activity levels are impacted by numerous contextual factors.[16]

There is a notable demand for a better understanding of the factors that influence obesity in adolescents to develop interventions to prevent and manage obesity in this crucial period of life. To the extent of our knowledge, no study has systematically reviewed the literature on the factors associated with the development of obesity focusing solely on adolescents. Thus, the purpose of the present systematic review was to identify and assess the peer-reviewed scientific literature on the behavioral, contextual and biological factors associated with obesity in adolescents aged 10 to 18 years.

## Methods

### Literature search strategy

PubMed and Scopus were systematically searched to identify relevant articles published between January 2000 and April 2018. The literature search was conducted between February and April 2018.

A combination of terms involving the population (adolescents), the exposure (biological, contextual and behavioral factors) and the outcome (overweight or obesity or body mass index (BMI)) was used. In PubMed, terms involving the study design (longitudinal, prospective or cohort or follow-up) were also combined with the previous terms, while in Scopus the limit to keyword feature was applied. The search terms are presented in Table 1.

**Table 1:** Search terms of the population, exposure and outcome used in PubMed and scopus.

Search framework	Search terms
Population	adolescent*; teen*; youth*; juvenil*.
Exposure	Behavioral factors; diet; eating habits; dietary intake; diet quality; food preferences; feeding practices; physical activity; sedentary behavior; sleep.
	Biological factors; genetic factors; heritability; twin*; parental overweight.
	Contextual factors; environmental factors; social environment; cultural environment; sociocultural environment; socioeconomic status; social influences; built environment; physical environment; school environment; household environment; food environment; family environment; neighborhood; parental influences.
Outcome	obes*; overweight; body mass index.

In addition, the reference lists of the selected studies and relevant reviews were hand searched to identify further relevant articles.

### Inclusion and exclusion criteria

For inclusion, articles were required to (1) be published in peer-reviewed journals between January 2000 and April 2018, (2) be published in English, (3) have a prospective cohort design, (4) include only adolescents between the ages of 10 and 18 years old, (5) to measure both, the outcome and the exposure of interest during the age range mentioned on point 4 (with the exception of infant feeding practices) and (6) use BMI or overweight/obesity (assessed using BMI) as the outcome measure.

No restriction on the method for assessing the exposure was considered.

Studies were excluded if they used reported height and weight and if subjects presented any medical conditions. Studies were also excluded if children and adolescents were examined as a total group and no separate analysis for the adolescent population existed.

### Quality assessment

The quality assessment of selected studies was performed by one reviewer (JN) using the Newcastle-Ottawa Scale[18] and revised by a second reviewer (AC). Studies were scored based on selection of study groups (four criteria), comparability of study groups (one criteria), and ascertainment of the outcome (three criteria). The final score ranged between 0 to 9 stars, with 9 representing the highest methodological quality. We considered the studies to be high, medium and low quality if the scores ranged between 7–9, 4–6 and 1–3, respectively. The quality assessment details for each study can be found in Table A in S2 Appendix.

## Results

### Study selection

**Fig 1:**
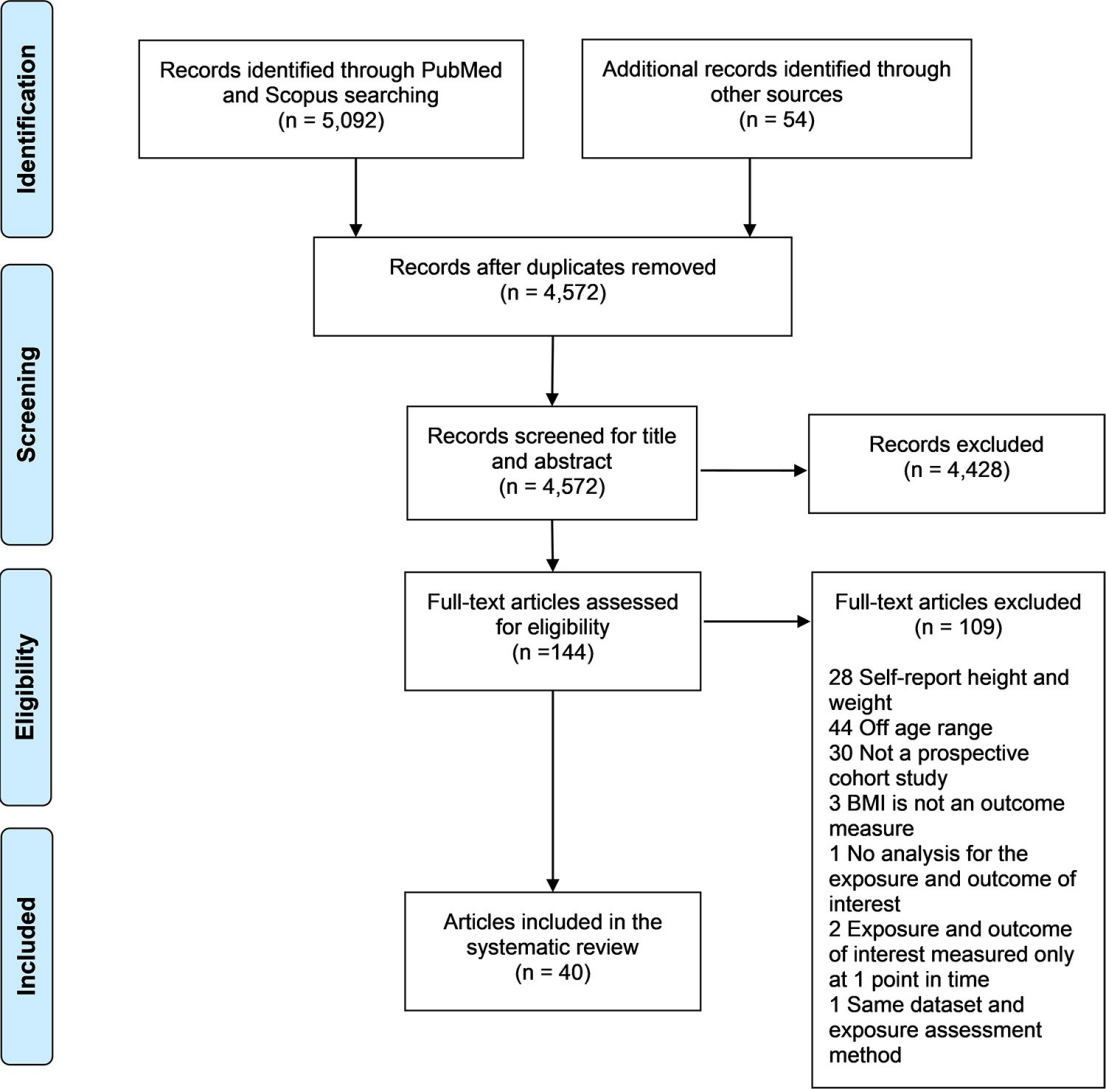
PRISMA flow diagram of study selection process.

The 5092 studies retrieved from both databases and the 54 studies retrieved from handsearching were exported to Mendeley (Version 1.17.13) where duplicates were excluded (n = 574). To identify relevant studies for the review a two-step process was used: first the information contained in the title and abstract was screened; and second if the abstracts did not provide enough information or were not available, the full-text articles were screened based on the inclusion-exclusion criteria previously described. One reviewer (JN) conducted the database search and study selection. However, if any doubts occurred during the selection process a second reviewer (AC) was consulted and a collaborative decision to include or exclude the study was made. Even though two studies[19,20] used the same sample to evaluate the relationship between neighborhood food environment and obesity, we decided to retain them as they measured the neighborhood food environment differently. We also decided to include one study[21] in which a minimal portion of the sample (n = 10) had height and weight measured by the parents using the investigators’ instructions. At the end of the selection process 40 articles were included in the review. A flow diagram of the selection process can be found in Fig 1.

### Characteristics of the studies included

The studies included in the present review were published between 2001 and 2017, with most of studies being published during or after 2010 (n = 33). Of the 40 studies included, the majority were conducted in North America (n = 15), followed by Europe (n = 10) and Australia (n = 8). The sample size ranged from 103[22] to 7090[19] participants and the follow-up period from 1[23,24] to 18 years[25]. Two studies included only females[26,27] and one study only males.[25]

Out of the 40 studies included, 26 [20–23,26,28–48] used BMI as their outcome, 6[24,27,49–52] used overweight/obesity, while 8 [19,25,53–58] studies used both measures. In 13 studies, body fat either expressed in percentage [25,26,28,29,33,34,38,43,44,53,56] or in kg[31,32] was used as the outcome alongside BMI or overweight/obesity. Furthermore, measures of fat mass index, fat free mass index, waist circumference (WC) and skinfold thickness were also concurrently used with BMI or overweight/obesity.

Regarding quality assessment, 21[23,25–29,31,33,35,36,40,41,43–45,48,51–55] studies were classified as high quality and 19[19–22,24,30,32,34,37–39,42,46,47,49,50,56–58] as medium quality. Studies final scores ranged from 4 to 8 stars.

A summary of all the studies can be found in Table 2 and the description of individual studies can be found in Table A in S3 Appendix.

**Table 2:** Summary of studies included in the review.

Exposure	Result’s summary	Result’s description	Quality score (Mean)
Dietary Intake			
Energy intake	PA*; 0	1 study[26]: higher energy intake was associated with higher BMI.	7.5
		1 study[23]: no association.	
Macronutrients	Mixed_†__;_ 0; 0*	1 study[28]: higher fiber intake was associated with lower BMI; higher carbohydrate consumption was associated with higher BMI.	7.7
		2 studies[23,26]: no association.	
GL	PA_†_; 0	1 study[28]: higher GL was associated with higher BMI.	8
		1 study[29]: no association.	
GI	0; 0	2 studies[28,29]: no association.	8
Dairy products	0; 0	2 studies[30,53]: no association.	6.5
SSB	PA; PA_ǂ_; PA; 0; 0; 0; 0	3 studies[31,32,54]: higher SSB consumption was associated with higher BMI.	6.9
		4 studies[23,28,33,55]: no association.	
Fruit/Vegetables	0; 0*; 0	3 studies[23,26,36]: no association.	7
Fast food	PA; 0; 0; 0	1 study[56]: higher fast food consumption was associated with higher BMI and odds of being obese.	6
		3 studies[32–34]: no association.	
Breakfast	PA; PA; 0; 0	2 studies: Breakfast skipping was associated with higher BMI[35] and odds of being overweight/obese.[49]	6
		2 studies[32,33]: no association.	
Infant feeding practices	0; 0**	2 studies[25,50]: no association.	6.5
Physical activity	NA*; NA; NA; PA; PA_†__;_ Mixed; 0; 0*; 0	3 studies: higher levels of physical activity were associated with lower risk of obesity in white girls[27], lower BMI [35] and lower odds of transitioning from overweight to obesity.[51]	6.7
		2 studies: higher levels of physical activity were associated with higher BMI trajectories.[36] Rates of decline in VPA were associated with declines in BMI.[38]	
		1 study[37]: higher physical activity fluctuation scores were associated with higher BMI in boys and with lower BMI in girls.	
		3 studies[23,26,39]: no association.	
Sedentary behavior	PA; PA; PA; 0; 0*; 0; 0	2 studies[35,40]: higher screen time was associated with higher BMI.	7.1
		1 study[51]: overweight 5^th^ graders were more likely to become obese if they watched more TV.	
		4 studies[23,26,39,41]: no association.	
Sleep	PA_†_; 0; 0; 0; 0	1 study[42]: improving sleep duration trajectories during adolescence was associated with higher BMI compared to those who always had adequate sleep duration.	6.8
		4 studies[24,36,43,44]: no association.	
Neighborhood food environment	Mixed_†__;_ Mixed	1 study[19]: greater quantity of supermarkets in a neighborhood was associated with lower BMI; greater quantity of limited-service restaurants in a neighborhood was associated with a higher BMI.	6
		1 study[20]: More types of food outlets in an area is associated with higher BMI; no association for counts of a particular type of food outlet per population and food environment indices with BMI.	
Family/home environment	Mixed; Mixed	1 study[45]: having non-married parents was associated with higher BMI; In boys, a higher number of sedentary items and time spent in MVPA by mothers was associated with higher BMI; In girls, a higher time spent in SB by mothers was associated with higher BMI, while a higher number of rules regarding SB and number of PA items was associated with lower BMI.	6.5
		1 study[57]: more frequent dinner consumption while watching TV was associated with higher BMI; less frequent breakfast intake was associated with higher odds of being overweight.	
School environment	PA; 0; 0; 0*	1 study[46]: higher vending machine availability was associated with higher BMI in Hispanic males and low-income Hispanic students.	6.8
		3 studies[26,55,58]: no association.	
SES	NA_†__;_ NA; PA; PA; 0	2 studies: lower SES was associated with higher odds of becoming obese[52] or remaining obese.[51]	6.4
		2 studies: higher deprivation was associated with higher BMI and risk of obesity[56]; Higher amount of money earned was associated with higher BMI.[35]	
		1 study[39]: no association.	
Parental restrictive feeding practices	0	1 study[21]: no association.	6
Self-control	NA	1 study[22]: higher self-control was associated with lower BMI.	6
Weight control	PA	1 study[52]: being in a diet to lose weight was associated with higher odds of becoming obese and ceasing to be obese.	7
Neighborhood environment	0	1 study[45]: no association.	7
Heritability of BMI	PA; PA	1 studies[51]: overweight 5^th^ graders were more likely to become obese if they had an obese parent.	6.5
		1 study[47]: Most of the variation in BMI was explained by genetic influences (additive and/or non-additive).	
Specific genes	PA	1 study[48]: Variation in the FTO gene and near the MC4R gene was associated with higher BMI.	7

NA–Significant negative association between the exposure and BMI or overweight/obesity; PA–Significant positive association; 0 –No association

*Study included only females

**Study included only males

† Association only in females

ǂ Association only in males.

Abbreviations: BMI–body mass index; GL–Glycemic Load; GI–Glycemic Index; SSB–sugar sweetened beverages; TV–television; VPA–vigorous physical activity; SES–socioeconomic status.

### Relation between behavioral factors and obesity

#### Dietary intake

In this review sixteen studies[23,26,28–36,49,53–56] examining the association between dietary intake and obesity during adolescence were included.

Cohen et al[26] examined whether changes in diet over a 2-year period were associated with changes in BMI and body fat in 265 girls and found that for every increase of 100 calories over the baseline consumed per day, girls gained 0.4 BMI percentiles points. However, no evidence of association between BMI and calories consumed from carbohydrates, protein and fat was found. Enes and Slater[23] found no significant associations between one-year change in BMI z-score and both, total energy and fat intake. However, a significant positive association was found for fatty food intake.

Gopinath et al[28] followed 1213 12 years old students for 5 years and reported that each 1-SD increase in dietary fiber intake was associated with a concurrent 0.44kg/m^2^ decrease in mean BMI in girls and a 1.45 cm decrease in WC in boys. Additionally, in girls each 1-SD increase in carbohydrates and dietary glycemic load was associated with an increase of 0.77kg/m^2^ in BMI. Conversely, Murakami et al[29] found that changes in glycemic load were not associated with concurrent changes in BMI or any other body composition measures in either boys or girls. In both studies[28,29], no association with baseline glycemic index and changes in BMI and percentage of body fat (%BF) were detected. However, Murakami et al[29] found that glycemic index was associated with increased change in fat mass index between the ages 12 and 15 years and that results regarding glycemic index and BMI (tertiles of baseline GI: 2.03(1.74, 2.32); 2.09 (1.80, 2.38) and 2.42 (2.13, 2.71) kg/m^2^; P_trend_ = 0.06) appear to point in the same direction.

Research regarding the consumption of dairy products was conducted by Bigornia et al[53] and Lin et al[30]. The first study[53] examined the association between dairy consumption at 10 years of age and risk of excess adiposity 3 years later. The authors found no association between total and reduced-fat dairy intake and risk of overweight or excess fat mass. Even though, in many cases the results did not achieve significance they showed inverse relations between dairy intakes and risk of excess adiposity, particularly for full-fat dairy products. However, the highest versus the lowest consumers of full-fat dairy products had smaller gains in BMI during follow-up and appeared to have a reduced risk of overweight at 13 years of age (OR: 0.65; 95% CI:0.40, 1.06; P = 0.19). The second study[30] using imputed data for 5,968 Chinese adolescents with BMI z-score, reported that neither non-milk dairy products nor milk consumption at 11 years was associated with BMI z-score at 13 years of age.

Three[31,32,54] of the seven[23,28,33,55] studies that looked at the association between sugar-sweetened beverages (SSB) and BMI found significant associations. Ludwig et al[54] reported that for each additional serving of SSB consumed, both BMI and odds of being obese increased. Likewise, results from *The Avon Longitudinal Study of Parents and Children* [31] showed that increased SSB consumption from ages 10 to 13 years was associated with higher BMI, WC and total body fat mass at 13 years, with the effects being strengthened among plausible dietary reporters Feeley et al[32] found a positive association between SSB consumption and both, BMI z-score and fat mass in South African males. It is important to note that Gopinath et al[28] found that girls who consumed one or more soft drinks/day compared to those who never/rarely consumed soft drinks at baseline had a greater %BF, BMI (3.20 versus 1.96 kg/m2, P = 0.01) and WC (10.00 versus 6.46 cm, P = 0.004) after 5 years. However, the trend was nonsignificant for BMI and WC. Additionally, Laska et al[33] reported that among males there was some evidence for a positive association between SSB consumption and both %BF and BMI (β = 0.27, P = 0.008). However, the last was not significant as α = 0.003125 was used to indicate statistical significance.

Studies regarding fast food consumption and obesity in adolescents have yield mixed results. Fraser et al[56] reported that increased consumption of fast food at age 13 years was positively associated with BMI SD scores, body fat percentage and being obese at age 15 years. However, three[32–34] other studies found no association between fast food or take-away food consumption and BMI.

Four studies[32,33,35,49] that examined the relationship between breakfast consumption and obesity in adolescence were identified. Elgar et al[35] found that skipping breakfast in Year 7 was positively associated with BMI in Year 11. Likewise, Wang et al[49] found that the odds of being overweight or obese was significantly higher for students that frequently skipped breakfast compared with those classified as double breakfast eaters. Two studies[32,33] found no association between breakfast consumption and BMI or fat mass.

No evidence of association was found between fruits/vegetables intake and BMI.[23,26,36]

#### Infant feeding practices

Two studies[25,50] that examined the relationship between breastfeeding and obesity were identified in our search.

Shields et al[50] examined the association between the duration of breastfeeding and being overweight or obese at 14 years of age in 3698 adolescents. They found that the risk of obesity was reduced for those breastfed for longer than six months, however when early life and 14 years confounders were taking into consideration the effect size diminished and lost statistical significance (OR:0.8, 95% CI: 0.5, 1.3). Even though no significant results were reported for risk of overweight, there was a trend for breastfeeding for less than 4 months to be associated with an increased prevalence of overweight at 14 years of age. Victora et al[25] explored the relationship between the duration of breastfeeding and several anthropometric measures in 18-year-old males. Neither the duration of total breastfeeding nor that of predominant breastfeeding showed consistent associations with any anthropometric or body composition measures. However, after adjustment for confounding variables (family income, maternal education at birth, maternal BMI, skin color, birth weight, gestational age, maternal smoking during pregnancy, and current behavioral variables -smoking, alcohol drinking, type of diet and physical exercise) a linear trend for a reduction in obesity with increasing duration of predominant breastfeeding remained.

#### Physical activity

This review identified nine articles[23,26,27,35–39,51] that examined the association between PA and obesity during adolescence.

Elgar et al[35] followed 355 adolescents aged 11 to 14 years for 4 years and found that the amount of time playing sports or exercising at baseline was negatively associated with changes in BMI, but it was not predictive of BMI at follow-up. Schuster et al[51] reported that in the bivariate analysis, overweight 5^th^ graders were less likely to become obese at 10^th^ grade if they performed more vigorous exercise. Furthermore, Bélanger et al[37] examined the association between the variation in the number of weekly moderate-to-vigorous PA (MVPA) sessions for 5 years and BMI, WC and skinfolds at follow-up in 756 adolescents. They found that in boys, higher PA fluctuation scores were associated with higher BMI and triceps skinfold thickness, while in girls higher PA fluctuation scores were associated with lower BMI, WC and skinfold thickness. Barnett et al[38] using the same sample as the previous study found that rates of decline in vigorous PA (VPA) were associated with declines in BMI during earlier adolescence in boys and slightly associated with declines in BMI during later adolescence in girls. However, MVPA and VPA were inversely associated with body fat percentage during earlier adolescence in girls and MVPA was inversely associated with body fat percentage during later adolescence in boys. de Souza et al[36] also contrary to what was expected, found that total PA had a positive association with girls’ BMI trajectories. Two studies[23,39] found no associations between PA and BMI.

Only two cohort studies[26,27] that used accelerometry to measure PA were identified. The first study[27] explored the associations between PA and 3 measurements of obesity (Centers for Disease Control and Prevention definition of obesity, the International Obesity Task Force reference BMI cut points for obesity in children and the sums of skinfold thickness—with the cohort ≥90th percentile as indicative of obesity) among 1148 black and white adolescent girls during a 2-year period. They reported a strong negative dose-response association between quartiles of accelerometer counts per day at 12 years of age and all the 3 measurements of obesity at age 14 years, but only for white girls. The second study[26] followed a cohort of adolescent girls for 2 years and found that every additional minute of increase in VPA per day was associated with a decline of body fat by approximately 0.1%. The association between BMI and VPA (β = -0.06, 95%CI: -0.28,0.16) pointed in the same direction.

#### Sedentary behavior

Seven studies[23,26,35,39–41,51] that examined the relationship between sedentary behavior (SB) and obesity were identified in our search.

Dumith et al[40] studied the effect of screen-time change (television (TV) + video game + computer) from age 11 to 15 years on several anthropometric measures at age 15 years and found that adolescents who increased their screen-time, had an increase in BMI, skinfold thickness and WC. Similarly, results from a cohort of Welsh adolescents[35] found that the number of hours per week watching TV or videos or playing computer games in Year 7 predicted BMI in Year 11. However, these sedentary activities did not predict change in BMI. Schuster et al[51] followed 3961 students for 5 years and reported that overweight 5^th^ graders who watched more TV were more likely to become obese. Conversely, Fletcher et al[41] followed a cohort of 285 Australian adolescents for approximately 2 years and found no evidence of direct or indirect prospective associations for TV viewing, objectively measured total SB and average sedentary bout duration at baseline with BMI z-score at follow-up. Cohen et al[26] also, found no evidence of association between change in objectively measured SB and changes in BMI percentile, in girls. Furthermore, two studies found no significant association between self-reported screen time and BMI.[23,39]

#### Sleep

This review identified five articles[24,36,42–44] that explored the association between sleep and obesity.

Schafer et al[42] assessed the relationship between sleep duration trajectories and BMI, fat mass and fat-free mass at 18 years old in 3974 adolescents. They found that girls who reported inadequate sleep duration (<8hours/day) at age 11, but adequate sleep duration (≥8hours/day) at 18, had an increase of 0.39 z-scores and 0.30 z-scores in BMI and fat mass index, respectively compared to those who always had adequate sleep duration. Araújo et al[43] studied 1171 adolescents at both 13 and 17 years of age and found that reported sleep duration at age 13 was inversely associated with BMI z-scores and %BF 17 years in boys. In girls sleep duration at age 13 was positively associated with the changes in BMI z-scores between 13 and 17 years of age. However, after adjusting for adiposity at baseline, the prior associations were no longer statistically significant. Roberts and Duong[24] examined the reciprocal association between sleep restriction (≥6hours/night) and obesity in 3134 adolescents followed over one-year period and found that sleep restriction did not increase the future risk of obesity. It is worth mentioning that the odds for short sleep all week was 1.85, but the confidence interval was relatively large (95% CI: 0.91; 3.73).Moreover, Lytle et al[44] combining data from two American studies, showed that there was no statistically significant relationship between change in total sleep and change in BMI or %BF over time in both genders. The only longitudinal relationship that approach statistical significance was a positive association between sleep and %BF in females (b = 0.268, 95% CI: -0.02, 0.56, P = 0.068). Similarly, de Souza et al[36] did not find any significant association between sleep duration and BMI trajectories in either boys or girls.

### Relation between contextual factors and obesity

#### Neighborhood food environment

This review identified two studies[19,20] that assessed the relationship between the neighborhood food store environment and obesity during adolescence.

Chen and Wang[19] found, amongst a nationally representative cohort of American students followed from 5^th^ to 8^th^ grade, that girls living in neighborhoods with 2 or more supermarkets (based on ZIP code) had a lower BMI by 0.6 kg/m^2^ three years later, in comparison with those living in places without supermarkets. Also, for girls a greater quantity of limited-service restaurants in a neighborhood was associated with both, a greater BMI and greater odds of being obese 3 years later. On the contrary, Shier and Sturm[20] used the same sample as the previous study and found no association between two measures of the neighborhood food environment (counts of a specific type of food outlet per 1000 population and food environment indices) and BMI percentile in 8^th^ grade or change in BMI percentile from 5^th^ to 8^th^ grade. Additionally, using a third measure of the neighborhood food environment (indicators for the presence of specific combinations of types of food stores) the authors reported that the existence of more types of food outlets in an area, including supermarkets, was associated with a higher BMI.

#### School environment

The association between school environment and obesity was evaluated in four studies[26,46,55,58].

Cunningham and Zavodny[55] investigated the relationship between the availability and purchases of sweetened beverages at school and obesity among 5^th^ and 8^th^ graders. They found that the availability of sweetened beverages at school did not lead to heavier weight or greater risk of overweight or obesity among American adolescents. The second study[46] examined the association between school vending machines availability and BMI changes from 5^th^ to 8^th^ grade among students’ subgroups. An increase in vending machine availability was associated with an increase of 0.46 and 0.40 units in BMI for Hispanic males and for low-income Hispanic students, respectively.

Wardle et al[58] examined the impact of school physical education (PE) on BMI and WC change over 5 years and found no significant effects of the number of PE classes scheduled on changes in BMI or among overweight and obese adolescents when compared to their normal-weight counterparts. However, after adjusting for confounder variables, boys in schools with three weekly PE sessions had a. smaller increase in WC over the 5 years than those in schools providing one or two sessions. Differences in girls were in the same direction but did not achieve statistical significance. Cohen et al[26] following a cohort of girls, also found no association between participation in school PE classes and BMI changes over 2 years. Surprisingly, a positive association between school PE and %BF was found.

#### Socioeconomic status

This review identified five studies[35,39,51,52,56] that explored the association between socioeconomic status (SES) and obesity.

Assunção et al[52] explored whether socioeconomic position (based on a list of 18 socioeconomic indicators) at age 11 years was associated with entry and exit from obesity between the ages of 11 and 15 years. They found that girls in the lowest tertile of socioeconomic position had a greater probability of becoming obese and that for boys, high socioeconomic position was a predictor of ceasing to be obese. Similarly, Schuster et al[51] reported that 5^th^ graders with a lower household education were more likely to remain and become obese from 5^th^ to 8^th^ grade, however after adjusting for confounders the last result was no longer significant (OR:1.83, 95% CI: 0.88, 3.79). Results from *The Avon Longitudinal Study of Parents and Children* [56] also showed a positive relationship between increasing deprivation and both increased BMI and odds of being obese at age 15 years. Contrarywise, Elgar et al[35] reported that most SES variables in Year 7 did not predict a change in BMI over 4 years, except for the amount of money earned, while Aires et al[39] found no association between SES based on parent’s education level and risk of being overweight/obese over a period of 3 years.

#### Other factors

Due to the limited number of studies, there was inconclusive evidence for parental feeding practices[21], self-control[22], weight loss dieting[52], home and neighborhood environment[45] and family environment[57].

### Relation between biological factors and obesity

#### Genetic factors

Three studies[47,48,51] that look at the association between genetic factors and obesity in adolescents were identified in the present review.

Cornes et al[47] examined whether there were qualitative and quantitative differences in genetic and environmental influences affecting BMI longitudinally in males and females using a sample of 1143 twin pairs at ages 12, 14 and 16 years. They found that most of the variation in BMI at all three ages was explained by additive and/or non-additive genetic influences for both genders. Liem et al[48] explored the association of common genetic variants near the *INSIG2*, in the *FTO* and near the *MC4R* genes and changes in BMI (3 time points) and skinfold thickness (2 time points) z-scores. Variation in the FTO gene was associated with BMI and sum of skinfolds longitudinally. In addition, variation near the *MC4R* gene was also associated with BMI. The last study[51] estimated the heritability of BMI between parents and their children and reported that overweight 5^th^ graders were more likely to become obese if they had an obese parent at baseline. Furthermore, obese 5^th^ graders were less likely to exit obesity if they had an obese parent.

## Discussion

This review included 40 prospective studies that explored the association between behavioral, contextual and biological factors and obesity in adolescents aged 10 to 18 years. There is evidence to support the association between genetic factors and SES with obesity. There was conflicting evidence for the contribution of dietary intake, PA, SB, sleep, food store environment and school food environment. Moreover, due to the limited number of studies identified regarding parental feeding practices, self-control, weight loss dieting, neighborhood environment and family/home environment, no reasonable conclusions could be drawn. No associations were identified for the remaining factors.

There is a consensus that obesity results from a long-term positive energy balance where dietary intake surpasses energy expenditure for metabolic processes and physical activity.[59] However, we found inconsistent evidence for the link between obesity and both, dietary intake and physical activity during adolescence. Similarly, two systematic reviews, one focusing on children and adolescents[60] and the other on adolescents and young adults[61], found that the longitudinal evidence regarding the association between dietary intake and body fat yield mixed results. In the current review, the inconsistent results concerning obesity and dietary intake can be, in part, explained by the diversity of methods used to assess diet alongside with the exclusive use of self-reported methods across all studies. Inaccurate dietary assessment may comprise a serious obstacle to understand the impact of dietary intake on the development of obesity. Therefore, continued efforts towards improving self-reported methods, as well as the use of technological advances to develop new methods to improve dietary assessment are crucial in the field of nutrition research.[62,63]

Regarding PA, most of the studies relied on self-reported measures and reported inconsistent results. However, it’s important to note that the two cohort studies[26,27] that included only girls and used accelerometry to assess PA found significant results in the expected direction; and that both studies[26,38] that looked at the association between %BF and PA found a protective, albeit weak relationship. Furthermore, differential effects of PA by gender[36–38] and across ethnicities[27] were found. Rauner et al[64] conducted a systematic review of cross-sectional and longitudinal studies and found that the evidence on the relationship between physical activity and overweight in adolescents was also inconsistent. On the other hand, Ramires et al[65] reviewed systematically 18 longitudinal studies on the association of PA and BF in adolescents and concluded that PA has some protective effect against BF with differences between the genders. Likewise, a systematic review by Pate et al[60] that examined several factors that predict the development of excessive fatness in children and adolescents concluded that there is enough evidence to support the negative association between objectively measured PA and excessive fatness. Given the controversial findings in the literature, it appears that the association between PA and the development of obesity in adolescence is not yet clearly understood. In the current review the mixed results might arise due to the inconsistency of methods used to assess PA, combined with an overreliance on self-reported measures. Additionally, failure to distinguish between the effect of different intensities of activity on adiposity may mitigate the possibility of detecting associations.[37]

SB can be defined as an immobile state of the body (sitting or reclining) during waking hours, resulting in energy expenditure close to the resting metabolic rate.[66,67] Research in this field has expanded exponentially since the early 2000s[67] and SB in youth has attracted attention as a potential risk factor for obesity, even though the evidence is far from conclusive.[68] In our review we found inconsistent evidence for the association between obesity and SB during adolescence. However, the studies that reported significant results[35,40,51] all pointed in the same direction, showing a small positive association between SB and BMI. On the other hand, the two studies[26,41] using objective measures of SB (accelerometry) yielded null associations. Similarly, a recent systematic review[67] including 29 reviews in children or adolescents under the age of 19 years concluded that claims for ‘clear’ associations between SB and adiposity in youth are premature or misguided, as there is limited evidence for causality and the associations range from small to very small. Once again, these findings may be due to the use of different measures of SB and the reliability on self-reported measures. All studies controlled for PA and most for some dietary factors, however future research in adolescents must take into account usual dietary intake, pubertal status and possibly sleep, as they are important potential confounders of any relationship between SB and adiposity.[67]

Indeed, sleep plays an important role in the growth, maturation and health of children and adolescents and sleep deprivation has emerged as a potential risk factor for the development of obesity.[44,69] However, we found conflicting evidence for the association between sleep and obesity during adolescence. Moreover, a gender difference appears to exist in the relationship between sleep and obesity during adolescence. Likewise, Chen et al[70] conducted a systematic review and meta-analysis and reported that findings from studies in adolescents were inconsistent and that, even though, most studies of adolescents gave significant results in the expected direction some reported no associations among girls. Contrarywise, a recent meta-analysis[71] of prospective studies concluded that short sleep duration is a risk factor for the development of obesity in infants, children and adolescents. However, these findings should be interpreted with caution as the analysis only included 3 cohort studies in adolescents. All studies included in this review were based on self-report measures of sleep duration and have not considered other sleep dimensions that could affect obesity. Only one study, addressed energy intake and PA as confounders. Therefore, more prospective studies using objective measures of sleep that consider lifestyle, behavioral and environmental confounding variables are necessary to understand the relationship between sleep and obesity in this age group.

Contextual factors exist at various levels, including the socio-environmental level that occur with families, peers and within other community environments (e.g. schools) and the physical environment (e.g. the access to and support for healthy eating and physical activity) that also operates at home, school and neighborhood environments.[16] However in the current review only studies regarding the SES, family/home environment, food and neighborhood environment, school environment, weight loss dieting, and self-control were identified and due to the limited number of prospective studies often identified for each factor, no consistent conclusions could be drawn.

The food environment constitutes one among several aspects of the obesogenic environment (obesity-promoting) and includes availability and accessibility to food as well as food advertising and marketing.[72] In the present review only two studies[19,20] regarding the food retail environment were identified. Recently, the retail food environment has been receiving increased attention due to its possible link with obesity-related behaviors[73] and it has been hypothesized that a greater access to fast food outlets and convenience stores is associated with a higher BMI, while a greater access to supermarkets might have the opposite effect due to its provision of numerous healthy products.[19,20] We found conflicting evidence regarding this hypothesis from two studies using the same sample. Additionally, a recent systematic review found no association between supermarket or grocery store availability and obesity in children, as the associations were null ≥90% of the time.[74] Further evidence using direct measures of food availability and data specifically designed to evaluate the association between food environment and obesity that considers additional confounding variables (e.g. pubertal status, dietary intake and PA) are necessary to understand the association between food environment and obesity in this age group.

School is a key social context in which children and adolescents spend approximately one-third of their waking time and in which many behaviors that can impact weight take place.[55,75] Children consume about 35 percent of their total daily energy intake from school meals[76] and spend approximately 50 percent of their daily energy expenditure at school (depending on the length of their school day).[77] However, despite its importance only four studies were identified that explored the association between the school environment and obesity during adolescence. This also occurred in Pate et al[60] review in 2013, where only one study regarding this subject was identified. Hence, further prospective studies are still required to understand the effects of the school environment on the development of obesity during adolescence.

SES may affect a population’s lifestyle, including their access to food and patterns of physical activity, which consequently will impact their energy balance.[78] In the current review four[35,51,52,56] of the five studies that look at the relationship between SES and obesity found an association, yet the direction of the association was not consistent across studies. Additionally, the strength of the association is weak in most studies. These findings are in accordance with the existing literature which shows that an association between obesity and SES exist; although the association seems to vary by gender, age, and country.[78] A comprehensive review that explored the impact of SES on obesity in children and adults was conducted in 1989. The authors observed that in developed countries, a clear negative association between SES and obesity was observed in women, while an inconsistent relationship in men, boys and girls existed. Contrarywise, in developing countries a positive relationship was observed in women, men, boys and girls.[79] A recent review focusing exclusively on the relationship between SES and childhood-adolescent weight status in rich countries reported that the relationship is mainly inverse (60.4%), while the positive relationship has nearly disappeared (1.1%). [80] Similarly, in our review only one study found a positive association between money earned and change in BMI in Wales[35]. The other study that showed a positive association points in the opposite direction since the exposure was the level of socioeconomic deprivation measured by the index of multiple deprivation (a non-linear measure of community socioeconomic status commonly used in the United Kingdom).[81] However in our review, the only study conducted in a developing country[52] reported that low-income girls were more likely to become obese when compared with their high-income counterparts. This might be explained by the fact that Brazil has been suffering a rapid social economic transition[78] and the burden of obesity has been shifting from the poor to the rich.[82] Hence, we conclude that an association between SES and obesity exists, however, the plethora of approaches and different measures of SES that were employed in the studies reviewed make it difficult to compare results across studies, and consequently understand the direction of the relationship between SES and obesity.

In what concerns biological factors, only three studies examining the relationship between genetic factors and obesity in adolescents were retrieved and all indicated that a positive association between genetics and obesity in this age group exists. Consistently, a systematic review by Pate et al[60] concluded that there is enough evidence to support the association between genetic factors and excessive fatness in children and adolescents. However, caution is necessary when considering our findings due to the limited number of studies and diversity of methods used to assess both, the exposure and outcome throughout the studies. Even though genetic factors acting in isolation are unlikely to explain the rapid increase of obesity prevalence during the last decades, it remains quite possible that genetic susceptibility coupled with environmental and behavioral factors might favor the development of obesity.[83,84] In addition, further biological factors that were not identified in our search, such as hormonal factors, endocrine disruptors and microbiological factors can also impact independently and/or synergistically the development of obesity.[85,86]

Regarding research quality, all studies included were classified as medium or high quality. Most studies performed poorly in “ascertainment of exposure” and “adequacy of follow-up” criteria. This occurred because some studies used non-validated self-reported measures to evaluate the exposure or were based on a very limited number of questions from a validated tool. Attrition bias was a frequent issue as, only approximately half of the studies had a follow up rate ≥80% or a description of those lost.

Overall, the 40 studies selected differed significantly in terms of the population, sample size and measurements used to assess both, the exposure and the outcome, which might, in part, explain the mixed results conveyed here. Additionally, several of the studies reviewed used already existing data (collected for other purposes other than studying the research question being considered) and examined the association between obesity and one factor at a time. Therefore, further prospective research that assesses multiple obesity risk factors simultaneously and uses state-of-art measures for both, the outcome and the exposure is necessary. Future research should also implement strategies to prevent loss to follow-up, as it often leads to bias and can severely compromise a study’s validity.[87,88]

Additionally, different statistical methods were used to analyze the relationship between the exposure and the outcome measure, with most studies using multivariate linear regression. However, as the exposure is often measured using different methods and metrics across the studies, the use of regression coefficients is not meaningful. Instead standardized regression coefficients should be used as an effect size estimate.[89] However, most studies fail to mention which measure was used, making it difficult to compare effects size across studies.

One strength of our review lies on the inclusion of studies that only used objectively measured weight and height, as adolescents’ self-reported anthropometric measures might lead to inaccurate estimation of overweight rates and there is bias by sex and weight status.[90,91] Nevertheless, self-reported data is valuable when objective measures are not available. The exclusive inclusion of prospective cohort studies represents a further strength, as this study design is considered the “gold standard” of observational research.[92] It is important to acknowledge that prospective cohort studies demonstrate only association[60] and in order to establish causal relationships experimental research is required.[93] However, not all obesity risk factors can be submitted to experimental manipulation, as researchers cannot randomize genes nor contextual factors such as income, education, food prices or social norms.[94] In addition, randomized controlled trials (the strongest study design for identifying causal relationships) are limited in the number of exposures they can evaluate at once[95] and their usefulness for studying the multifactorial etiology of obesity might be limited.

This review is not without limitations. First, it is important to note that this review comprises 40 studies, each one with their own limitations. Secondly, the exclusive use of English-language studies published during or after the year 2000 might have led to the exclusion of relevant evidence; Thirdly, the review might be prone to publication bias, as only the peer-reviewed literature was searched. Lastly, the use of BMI as the outcome measure constitutes an additional limitation, as BMI does not measure body fat directly nor does it distinguish fat mass from lean mass.[96,97] However, BMI is the most widely used and accepted measure of obesity and has shown strong correlation with gold standard measures of body fat.[96]

The authors would also like to acknowledge that even though no associations were found between breastfeeding or fruit/vegetables and obesity in adolescents, both exposures have been associated with numerous health benefits[98,99] Thus, promoting and supporting exclusive breastfeeding in the first 6 months of life[100] and the consumption of more than 400 grams of fruit and vegetables per day[101] should remain public health priorities.

## Conclusions

In the current review we found a positive consistent association between genetic factors and obesity during adolescence, however these findings should be interpreted with caution due to the heterogeneity and small number of studies identified. Furthermore, there is evidence to support the association between SES and obesity. For the remaining factors the evidence was either conflicting, limited or non-existent. Hence, the association between most risk factors and obesity during adolescence remains unclear. We also highlighted the heterogeneity of the studies included in this review, particularly regarding the methods used to assess the exposure and the outcome, which makes comparisons across studies difficult. Therefore, these findings reinforce the critical need for further prospective studies that examine the effect of multiple factors concurrently on obesity using state-of-art measures. This is imperative, as a profounder understanding of the multifactorial contributors to obesity and possible interactions between them will aid in the development of effective strategies and interventions to manage and prevent obesity during adolescence.

## Supporting information

S1 Appendix Search strategy used in PubMed. (DOCX)

S2 Appendix Quality assessment of the prospective cohort studies included in the present systematic review. (DOCX)

S3 Appendix Characteristics of the 40 studies included in the systematic review. (DOCX)

S4 Appendix PRISMA checklist. (DOC)
