# Supplementary material for: Behavioral, contextual and biological factors associated with obesity during adolescence: A systematic review
Source: PLoS One. 2019 Apr 8;14(4):e0214941. doi: 10.1371/journal.pone.0214941 (PMC6453458; doi:10.1371/journal.pone.0214941)
Supplement: S1 Appendix — (DOCX) [file pone.0214941.s001.docx]

**S1 Appendix. Search strategy used in PubMed**

| **Keywords** | | |
| --- | --- | --- |
| **Population** | (adolescen*[Title/Abstract] OR teen*[ Title/Abstract] OR youth*[ Title/Abstract] OR juvenil*[ Title/Abstract]) | AND |
| **Exposure** | (environmental factors[Title/Abstract] OR contextual factors[Title/Abstract] OR social environment[Title/Abstract] OR cultural environment[Title/Abstract] OR sociocultural environment[Title/Abstract] OR socioeconomic status[Title/Abstract] OR social influences[Title/Abstract] OR built environment[Title/Abstract] OR physical environment[Title/Abstract] OR school environment[Title/Abstract] OR household environment[Title/Abstract] OR food environment[Title/Abstract] OR family environment[Title/Abstract] OR neighborhood[Title/Abstract] OR parental influences[Title/Abstract] OR behavioral factors[Title/Abstract] OR diet[Title/Abstract] OR eating habits[Title/Abstract] OR dietary intake[Title/Abstract] OR diet quality[Title/Abstract] OR food preferences[Title/Abstract] OR feeding practices[Title/Abstract] OR physical activity[Title/Abstract] OR sedentary behavior[Title/Abstract] OR sleep[Title/Abstract] OR biological factors[Title/Abstract] OR genetic factors[Title/Abstract] OR heritability[Title/Abstract] OR twin*[Title/Abstract] OR parental overweight[Title/Abstract]) | AND |
| **Outcome** | (obes*[Title/Abstract] OR overweight[Title/Abstract] OR body mass index[Title/Abstract]) | AND |
| **Type of study** | (cohort[Title/Abstract] OR prospective stud*[Title/Abstract] OR longitudinal stud*[Title/Abstract] OR follow-up[Title/Abstract] | AND |
| **Filters** | (Journal Article[ptyp] AND ("2000/01/01"[PDat]: "2018/04/31"[PDat]) AND Humans[Mesh] AND English[lang]) | AND |
