# Supplementary material for: Behavioral, contextual and biological factors associated with obesity during adolescence: A systematic review
Source: PLoS One. 2019 Apr 8;14(4):e0214941. doi: 10.1371/journal.pone.0214941 (PMC6453458; doi:10.1371/journal.pone.0214941)
Supplement: S2 Appendix — (DOCX) [file pone.0214941.s002.docx]

**S2 Appendix.** **Quality assessment of the prospective cohort studies included in the present systematic review**

| **First author, year** | **Representativeness of the exposed cohort** | **Selection of the unexposed cohort** | **Ascertainment of exposure** | **Outcome of interest not present at start of study** | **Control for important factor or additional factors** | **Outcome assessment** | **Follow-up long enough for outcomes to occur** | **Adequacy of follow-up of cohorts** | **Total score** |
| --- | --- | --- | --- | --- | --- | --- | --- | --- | --- |
| Gopinath, 2013 | **¯** | **¯** | **¯** | --- | **¯¯** | **¯** | **¯** | **¯** | 8 |
| Laska, 2012 | **¯** | **¯** | **¯** | --- | **¯¯** | **¯** | **¯** | --- | 7 |
| Lin, 2012 | **¯** | **¯** | **¯** | --- | **¯¯** | **¯** | --- | --- | 6 |
| Bigornia, 2014 | **¯** | **¯** | **¯** | --- | **¯¯** | **¯** | **¯** | --- | 7 |
| Dumith, 2012 | **¯** | **¯** | **¯** | --- | **¯¯** | **¯** | **¯** | **¯** | 8 |
| Fletcher, 2017 | **¯** | **¯** | **¯** | --- | **¯¯** | **¯** | **¯** | **¯** | 8 |
| Fraser, 2012 | **¯** | **¯** | --- | --- | **¯¯** | **¯** | **¯** | --- | 6 |
| Macfarlane,2009 | **¯** | **¯** | --- | --- | **¯** | **¯** | **¯** | **¯** | 6 |
| Lytle, 2014 | **¯** | **¯** | **¯** | --- | **¯¯** | **¯** | **¯** | **¯** | 8 |
| Chen, 2016 | **¯** | **¯** | **¯** | --- | **¯** | **¯** | **¯** | --- | 6 |
| Crawford, 2010 | **¯** | **¯** | **¯** | --- | **¯** | **¯** | **¯** | **¯** | 7 |
| Enes, 2013 | **¯** | **¯** | **¯** | --- | **¯¯** | **¯** | --- | **¯** | 7 |
| Feeley, 2012 | **¯** | **¯** | --- | --- | **¯** | **¯** | **¯** | --- | 5 |
| Duckworth, 2010 | **¯** | **¯** | **¯** | --- | **¯** | **¯** | **¯** | --- | 6 |
| Gopinath, 2015 | **¯** | **¯** | --- | --- | **¯¯** | **¯** | **¯** | --- | 6 |
| Barnet, 2013 | --- | **¯** | **¯** | --- | **¯** | **¯** | **¯** | **¯** | 6 |
| Shields, 2006 | **¯** | **¯** | --- | --- | **¯** | **¯** | **¯** | **¯** | 6 |
| Victora, 2003 | **¯** | **¯** | --- | --- | **¯¯** | **¯** | **¯** | **¯** | 7 |
| Murakami, 2014 | **¯** | **¯** | **¯** | --- | **¯¯** | **¯** | **¯** | **¯** | 8 |
| Bigornia, 2015 | **¯** | **¯** | **¯** | --- | **¯¯** | **¯** | **¯** | --- | 7 |
| Schafer, 2016 | **¯** | **¯** | --- | --- | **¯** | **¯** | **¯** | **¯** | 6 |
| Roberts, 2015 | **¯** | **¯** | **¯** | --- | **¯** | **¯** | --- | **¯** | 6 |
| Araújo, 2012 | **¯** | **¯** | --- | --- | **¯¯** | **¯** | **¯** | **¯** | 7 |
| Bélanger, 2011 | --- | **¯** | **¯** | --- | **¯¯** | **¯** | **¯** | --- | 6 |
| Elgar, 2004 | **¯** | **¯** | **¯** | --- | **¯¯** | **¯** | **¯** | --- | 7 |
| Assunção, 2012 | **¯** | **¯** | **¯** | --- | **¯** | **¯** | **¯** | **¯** | 7 |
| Wang, 2017 | **¯** | **¯** | --- | --- | **¯** | **¯** | **¯** | --- | 5 |
| Wardle, 2007 | **¯** | **¯** | **¯** | --- | **¯** | **¯** | **¯** | --- | 6 |
| White, 2012 | --- | **¯** | **¯** | --- | **¯¯** | **¯** | **¯** | **¯** | 7 |
| Cunningham, 2011 | **¯** | **¯** | --- | --- | **¯¯** | **¯** | **¯** | **¯** | 7 |
| O’Hara, 2015 | **¯** | **¯** | --- | --- | **¯¯** | **¯** | **¯** | --- | 6 |
| Shier, 2012 | **¯** | **¯** | **¯** | --- | **¯** | **¯** | **¯** | --- | 6 |
| Cornes, 2007 | --- | **¯** | **¯** | --- | **¯** | **¯** | **¯** | --- | 5 |
| Liem, 2010 | **¯** | **¯** | **¯** | --- | **¯** | **¯** | **¯** | **¯** | 7 |
| Cohen, 2014 | **¯** | **¯** | **¯** | --- | **¯¯** | **¯** | **¯** | **¯** | 8 |
| Schuster, 2014 | **¯** | **¯** | **¯** | --- | **¯¯** | **¯** | **¯** | **¯** | 8 |
| Aires, 2010 | **¯** | **¯** | --- | --- | **¯** | --- | **¯** | --- | 4 |
| de Souza, 2015 | **¯** | **¯** | **¯** | --- | **¯¯** | **¯** | **¯** | --- | 7 |
| Ludwig, 2001 | **¯** | **¯** | **¯** | --- | **¯¯** | **¯** | --- | **¯** | 7 |
| Campbell, 2010 | **¯** | **¯** | --- | --- | **¯** | **¯** | **¯** | **¯** | 6 |
