# Supplementary material for: Behavioral, contextual and biological factors associated with obesity during adolescence: A systematic review
Source: PLoS One. 2019 Apr 8;14(4):e0214941. doi: 10.1371/journal.pone.0214941 (PMC6453458; doi:10.1371/journal.pone.0214941)
Supplement: S3 Appendix — (DOCX) [file pone.0214941.s003.docx]

**S3 Appendix. Characteristics of the 40 studies included in the systematic review.**

| **Author, Year**  **Country** | **Sample** | **Exposure** | **Exposure assessment** | **Outcome** | **Significant findings** | **Adjusted Variables** |
| --- | --- | --- | --- | --- | --- | --- |
| Enes and Slater, 2013  Brazil | Probalistic sample of students from public schools of Piracicaba, Brazil (N=299)  Mean age at baseline: 11.8 years  Follow-up: 1 year | PA, screen time, energy, fat, fatty foods, fruits and vegetables, sugar sweetened drinks, sweetened fruit juices and sweets and added sugar. | PA: validated questionnaire.  Dietary assessment: validated semi-quantitative FFQ. | One-year change in BMI z-score | An increased consumption of fatty foods (β=0.04, P=0.04) and natural sweetened fruit juices (β=0.05, P=0.03) was positively associated with an increase in BMI z-score. | Baseline BMI z-score, interval between study interviews, gender, sexual maturation and baseline age |
| Cohen et al, 2014  USA | Adolescent female subsample from the control group of the Trial of Activity for Adolescent Girls (TAAG) (N=265)  8^th^ grade at baseline  Follow-up: 2 years | Moderate to vigorous PA, sedentary behavior, physical education classes, total daily calories, calories from CHO, fat and protein, daily servings of fruits and vegetables, calories from SSB and snacks | PA and sedentary behavior: accelerometry  PE classes: Questionnaire  Dietary assessment: Validated FFQ (Youth/Adolescent Questionnaire) | Change in BMI percentile | Increased daily calories intake was associated with an increase in BMI percentile (β=0.004, p<0.01) | Age, race/ethnicity, mother’s education and neighborhood households in poverty. |
| Gopinath et al, 2013  Australia | Stratified random cluster sample from the Sydney Childhood Eye Study (N=856)  Age at baseline: 12 years old  Follow-up: 5 years | Glycemic Index, Glycemic Load of diets and intakes of carbohydrates, sugars, fiber and the main carbohydrates containing food groups (e.g. soft drinks) | Dietary assessment: Validated semiquantitative FFQ. | Change in mean BMI (kg/m^2^) from age 12 to 17 years | In girls an increase in dietary GL was associated with an increase in BMI (β=0.77, p=0.01); Dietary fiber intake was associated with a decrease in mean BMI in girls (β= -0.44, p=0.02). | Age, ethnicity, parental education, exposure to passive smoking, change in energy intake and height, screen time and PA. |
| Murakami et al, 2014  United Kingdom | Northern Ireland Young Hearts Study (N=426)  Age at baseline: 12 years old  Follow-up: 3 years | Dietary glycemic index and glycemic load | Dietary assessment: diet history with open-ended interview | Change in BMI (kg/m^-2^) and BMI z-score between ages 12 and 15 years. | There was no significant association of baseline/changes in GI or GL with changes in BMI. | Gender, pubertal status at baseline, maternal BMI at baseline, PA score at baseline and follow-up, ratio of energy intake to estimated energy requirement at baseline, baseline body composition values, baseline intakes of protein and dietary fiber and smoking status at follow-up. For the GI analysis baseline intake of total fat was also included. |
| Bigornia et al, 2014  United Kindgom | The Avon Longitudinal Study of Parents and Children (N=2455)  Age at baseline: 10 years old  Follow-up: 3 years | Dairy consumption (total, full and reduced-fat dairy) | Dietary assessment: 3-day dietary records | Overweight status at 13 years of age and 3-years change in BMI (kg/m^2^). | The highest vs the lowest quartile of total dairy consumers at 10 years of age did not have an increase of overweight (OR=0.69, 95% CI=0.41, 1.15; p=0.24) at 13 years of age.  The highest vs the lowest consumers of full fat products had smaller gains in BMI at follow up [2.5kg/m^2^(95% CI 2.2-2.7) vs 2.8 kg/m^2^(95% CI=2.5, 3.0); p<0.01] | Age and height at 10y, gender, total dairy at 13 y, adiposity at 10y, maternal education and overweight status, PA at 13y, pubertal status at 13y, dieting at 13y, age-10-y intakes of fruit juice, fruit and vegetables, total fat, total protein, SSB, fiber and cereal, dietary reporting errors at 13y and total dairy intakes. |
| Lin et al, 2012  China | The Hong Kong “Children of 1997” birth cohort(N=5968)  Age at baseline: 11 years old  Follow-up: 2 years | Milk and non-milk dairy products consumption | Dietary assessment: FFQ | BMI z-score at 13 years | Milk or other dairy products consumption at 11 years of age were not associated with BMI z-score at age 13 years. | Gender, BMI at 11 years old, birth order, maternal age, mother’s birth place, highest parental education, interaction of mater’s birth place and education, PA, vegetable, fruit and soft drink consumption. |
| Ludwig et al, 2001  USA | Participants from 5 randomly assigned control schools of the Planet Health intervention and evaluation project (N=548)  Mean age at baseline: 11.7 years  Follow-up: 19 months | Sugar sweetened drinks consumption | Dietary assessment: Youth FFQ (adapted and validated) | Change in BMI (kg/m^2^) and obesity incidence from baseline to follow-up. | For each additional serving of sugar-sweetened drink consumed, both BMI (mean 0.24 kg/m^2^;95% CI= 0.10, 0.39; p=0.003) and odds of being obese (OR=1.60; 95% CI=1.14, 2.24; p=0.02) increased.  Baseline consumption of SSB was also independently associated with change in BMI (mean 0.18kg/m^2^ for each daily serving; 95% CI 0.09-0.27; p=0.02) | Baseline anthropometrics (BMI and triceps-skinfold thickness), demographics (age, gender and ethnicity), % energy from fat and adjusted fruit juice intake at baseline and change in these variables from baseline to follow up, baseline self-reports of menarcheal status, PA, TV viewing, total energy intake. |
| Bigornia et al, 2014  United Kindgom | The Avon Longitudinal Study of Parents and Children (N=2455)  Age at baseline: 10 years old  Follow-up: 3 years | SSB intake | Dietary assessment: 3-day dietary records | BMI (kg/m^2^) at age 13 years. | Increased SSB consumption from ages 10 to 13 years was associated with higher BMI at 13 years (standardized β=0.028, p=0.03), with the effects being strengthened among plausible dietary reporters (standardized β=0.074, p<0.001) | Gender, baseline age, height, adiposity, SSB at baseline, PA at follow-up, pubertal status at follow-up, maternal overweight/obesity status, maternal education, dieting at follow-up, fruit juice and vegetable and total fat intakes from ages 10 to 13 years and dietary reporting errors at follow-up. |
| Feeley et al, 2012  South Africa | The birth to twenty cohort (N=1298)  Age at baseline: 13 years  Follow-up:4 years | Breakfast consumption, snaking while watching TV, eating main meal with family, lunch box use, number of tuck shop purchases, fast-food, confectionary and SSB consumption. | Dietary assessment: interviewer-assisted questionnaires | BMI Z-score at 17 years of age (2^nd^ follow-up). | In males, SSB consumption was positively associated with BMI Z-score (β=0.044, 95% CI=0.022, 0.067; p<0.01). | Change in SES between birth and 12 years of age. |
| Laska et al, 2012  USA | Combination of two longitudinal cohort studies: (1) The Identifying Determinants of Eating and Activity and (2) Etiology of Childhood Obesity (N=693)  Mean age at baseline: 14.6 years of age  Follow-up: 2 years | Dietary behaviors (SSB, diet soda, breakfast and fast food consumption) | Dietary assessment: telephone-administered 24-h dietary recalls; fast-food intake was assessed via a survey item. | Change in BMI (kg/m^2^) from baseline to follow-up. | SSB, diet soda, breakfast and fast food consumption were not associated with BMI. | PA at baseline, puberty, race, parental education, school lunch, age, study and total energy intake at baseline. |
| Cunnigham and Zavodny, 2011  USA | Multistage probability sample of the Early Childhood Longitudinal Study, Kindergarten Class (N= 6128)  School year at baseline: 5^th^ grade  Follow-up: 3 years | Access to the sale of SSB at school and total consumption of SBB | Access to the sale of SSB at school and SSB consumption: food consumption questionnaire. | Changes in BMI z-scores and overweight/obesity status from 5^th^ to 8^th^ grade. | There was no significant relationship between administrator reports of SSB availability and children’s BMI z-scores or odds of being overweight or obese.  No evidence that SSB consumption affects children’s weight. | Gender, age, race/ethnicity, changing school, activity in 5^th^ grade, SES, household and school characteristics. |
| Fraser et al, 2012  United Kingdom | Cohort from the Avon Longitudinal Study of Parents and Children(N=4837)  Age at baseline: 13 years  Follow-up: 2 years | Fast-food consumption | Dietary assessment: a single question from FFQ completed by the mother  Deprivation: index of multiple deprivation (IMD 2007) assigned to each participant. | BMI SD score and obesity status at age 15 years | Fast food consumption at age 13 years was associated with higher BMI SD score (β=0.08, 95% CI=0.03, 0.14) and higher odds of being obese (OR=1.23, 95% CI=1.02, 1.49) at age 15 years.  Increased deprivation was associated with higher BMI SD score (β=0.0044, 95% CI=0.002, 0.007) and with being obese (β=0.0150, 95% CI= 0.009, 0.02) | Gender, deprivation and PA levels at baseline. |
| Gopinath et al, 2016  Australia | Stratified random cluster sample from the Sydney Childhood Eye Study (N=699)  Age at baseline: 12 years old  Follow-up: 5 years | Take away food consumption | Dietary assessment: a single question from a validated semiquantitative FFQ. | Mean BMI (kg/m^2^) at the age of 17 years. | No significant associations were found between take away food consumption at the age of 12 years and BMI at the age of 17 years. | Age, gender, ethnicity, parental education, parental employment, PA and energy intake. |
| Elgar et al, 2005  United Kingdom | Cohort of Welsh adolescents from the Health Behavior of School-aged Children (N=355)  Mean age at baseline: 12.3 years (Year 7)  Follow-up: 4 years | PA, Sedentary Behavior, Breakfast consumption and SES | HBSC questionnaire. | BMI (kg/m^2^) at follow-up (Year 11) and change in BMI from baseline to follow-up | Sedentary behavior (β=0.19, p<0.01) and breakfast skipping (β=0.13, p<0.05) at baseline predicted BMI at follow-up. PA (β=˗0.08, p<0.05) and amount of money earned (β=0.09, p<0.05) predicted a change in BMI over time. | Age, gender, number of parents, family size, SES, meal skipping and snacks per day. |
| Wang et al, 2016  USA | Twelve schools randomly selected from a medium size urban school district (N=513 in 5^th^ grade, N=553 in 6^th^ grade and 468 in 7^th^ grade)  School year at baseline: 5^th^ grade  Follow-up: 2 years | Breakfast location patterns (frequency and place of breakfast consumption) | Breakfast location patterns: two questions from the student surveys. | Overweight /obesity changes over time (3 time periods assessment: 5^th^, 6^th^ and 7^th^ grade) | Increased odds of being overweight/obese among frequent breakfast skippers compared to double breakfast eaters (AOR:2.66, 95% CI=1.67, 4.24) | Gender, race/ethnicity, school and study year. |
| De Souza et al, 2015  Portugal | Randomly selected adolescents from four age cohorts from the Oporto Growth, Health and Performance study (N=6894)  Age at baseline:10, 12, 14 and 16 years  Follow-up: 2 years | Total PA, usual sleep duration and fruit/vegetables consumption. | Total PA: Baecke questionnaire (validated).  Usual sleep duration: questionnaire.  Fruit/vegetable intake: FFQ adapted from the ISCOLE and HBSC questionnaire. | BMI (kg/m^2^) trajectories from 10 to 18 years. | Total PA showed a positive association with girls’ BMI trajectories (β=0.10±0.03; p=0.001). | Physical Fitness tests, peak height velocity, total PA, sleep habits, fruit/vegetables intake. |
| Shields et al, 2006  Australia | Subsample of the Mater-University Study of Pregnancy cohort (N=3698)  Age at baseline: 6 months  Follow-up:13.5 years | Breastfeeding | 1 question from the maternal questionnaire at 6 months. | Overweight/obesity status at 14 years of age | Breastfeeding for less than 4 months had no effect on either obesity or overweight however a trend was found for increased prevalence of overweight at 14 years. | Small-for-gestational-age status, parents BMI, maternal age, maternal education level, hours spent watching TV at follow-up and attrition risk category (based on: maternal age <20 years, low income, low maternal education and non-white race). |
| Victora et al, 2003  Brazil | Male subsample from the 1993 Pelotas (Brazil) Birth Cohort Study (N= 2250)  Follow-up:18 years | Breastfeeding duration | Interview (mothers) | BMI (kg/m^2^) and overweight/obese status at 18 years of age. | There was a >50% reduction in obesity among participants breastfed for 3 to 5 months compared with all other breastfeeding categories (P=0.007) and a linear decreasing trend in obesity with increasing duration of predominant breastfeeding (P=0.03). | Family income and maternal education at birth, maternal BMI, skin color, birth weight, gestational age, maternal smoking during pregnancy, and current behavioral variables (smoking, alcohol drinking, type of diet and physical exercise). |
| Schuster et al, 2014  USA | The Healthy Passages study (N=3961)  Mean age at baseline: 11.1 years  Follow-up: 5 years | Parent BMI, child fast-food and soda consumption, vigorous exercise, TV viewing and sociodemographic characteristics. | Parent BMI: height and weight measured by trained and certified interviewers.  Remaining exposures: computer-assisted personal interviews and audio-assisted self-interviews | Exit from and entry (from overweight) into obesity between 5^th^ and 10^th^ grade | In the multivariate analysis, those who had lower household education (aOR:0.60; 95% CI: 0.42, 0.86, p=0.006) were less likely to exit obesity; Those who had an obese parent (aOR:2.79, 95% CI: 1.58, 4.91, p<0.001) or watched more TV (aOR:1.26 per 10 hours; 95% CI: 1.03,1.53; p=0.02) were more likely to become obese.  In the bivariate analysis, overweight 5th graders were less likely to become obese (OR:0.90 per day, 95% CI:0.82,0.99, P=0.04) if they performed more vigorous exercise. | Child BMI percentile at baseline |
| Bélanger et al, 2011  Canada | The Nicotine Dependence in Teens cohort study (N=756)  Age at baseline: 12-13 years  Follow-up: 5 years | PA fluctuation score | 7-day PA recall questionnaire based on the Weekly Activity Checklist | Changes in BMI (kg/m^2^) between baseline and the end of follow-up | A positive association between PA fluctuation and BMI was found among boys (β=0.12, 95% CI= 0.02, 0.21) and among girls a negative association between PA fluctuation and BMI was found (β= ˗0.12, 95% CI= ˗0.20, -0.03). | Baseline body fat, average number of PA sessions per week over the 5 years of the study, fruit/vegetable consumption, junk food consumption, number of siblings, family status and parental education. |
| Barnettt et al, 2013  Canada | The Nicotine Dependence in Teens cohort study (earlier adolescence period N=840; later adolescence period N=760)  Age at baseline: 12-13 years  Follow-up: 4.6 years | PA trajectories (VPA and MVPA) during secondary school | 7-day PA recall questionnaire based on the Weekly Activity Checklist | Change in BMI (kg/m^2^) during earlier adolescence (survey cycle 1 and 12) and during later adolescence (survey cycle 12 and 19) | Rates of decline in VPA were associated with declines in BMI in earlier adolescence among boys (β= -0.119, 95% CI= -0.084, 0.011) and marginally associated with declines in BMI in later adolescence in girls (β= -0.181, 95% CI= -0.392, 0.029). In girls, rates of decline in MVPA were also marginally associated with declines in BMI during later adolescence (β= -0.111, 95% CI= -0.241, 0.020). | Age and adiposity at the beginning of each follow-up time. |
| Aires et al, 2009  Portugal | Sample from a middle and high public school in a suburban setting(N=345)  Age at baseline: 11-16 years  Follow-up: 3 years | PA index, Screen time and SES (parental education level) | PA index/ screen time: questionnaire | Three-years changes in BMI (kg/m^2^) corrected for age and gender. | PA index, screen time and SES showed no association with BMIc. | Mother’s education, year, PA index, physical fitness tests, commuting to and from school and screen time. |
| White and Jago, 2012  USA | The National Heart, Lung, and Blood Institute Growth and Healthy Study (N=1148)  Age at baseline: 12 years  Follow-up: 2 years | PA | Accelerometry | Obesity status at the age of 14 years. | There was a strong negative dose-response association between quartiles of accelerometer counts per day at 12 years of age and obesity at 14 years of age in white but not in black girls (p<0.001 for BMI interaction and p=0.06 for sums of skinfolds interaction) | BMI of percentage of body fat at baseline, age, height, height squared, highest level of parental education and total annual household income, hours of TV viewing per week, self-reported PA metabolic equivalent tasks per week and total caloric intake per week and pubertal status at baseline and follow-up. |
| Dumith et al, 2012  Brazil | The 1993 Pelotas (Brazil) Birth Cohort Study (N=4,218)  Mean age at baseline: 11.3 years  Average follow-up: 3.4 years | Screen time | Face-to-face interview | BMI (kg/m^2^) at 15 years of age. | Adolescents that increased their screen time from 11 to 15 years of age had a higher BMI at age 15 years (β=0.06, 95% CI= 0.01, 0.10) | Screen time at baseline, gender, skin color, assets index and maternal schooling. |
| Fletcher et al, 2017  Australia | The Nepean Kids Growing-Up Study (N=259)  Age at baseline: 12-15 years  Follow-up: 2 years | Tv viewing, total sedentary time and average sedentary bout duration | Tv viewing: self-report questionnaire  Sedentary time: accelerometer | BMI z-score at follow-up | No significant longitudinal total or direct associations were observed for TV viewing total sedentary time and average sedentary bout duration with BMI z-score. | Age at baseline, gender, maternal education, pubertal status, accelerometry-measured moderate-to-vigorous PA at baseline and BMI a-score at baseline.  Mediating variables: dietary intake variables. |
| Schafer et al, 2016  Brazil | The 1993 Pelotas (Brazil) Birth Cohort Study (N=3974)  Age at baseline:11 years of age  Follow-up: 7 years | Sleep duration | Based on two questions: “what time do you usually fall asleep on weekdays?” and “what time do you usually wake up on weekdays?”. | BMI z-scores  Measured at follow-up 18 years of age | Girls who reported inadequate sleep duration at 11 years of age, but adequate sleep duration at 18 years, on average experienced an increase in BMI (β=0.39 z-score, 95% CI= 0.13, 0.65) compared to those who had adequate sleep duration at both time points. | Family income, maternal education, maternal skin color, maternal age ta birth, gestational weight gain, pregnancy smoking, pregnancy alcohol consumption, birth order, type of delivery, birth weight, PA and screen time at baseline. |
| Araújo et al, 2012  Portugal | Urban population-based cohort study (EPITeen) (N=1171)  Age at baseline: 13 years  Follow-up: 4 years | Sleep duration | Self-administered questionnaire (usual bedtimes and wake-up times on week-days) | BMI z-scores at 18 years of age and change in BMI z-scores from 13 years to 18 years of age | Sleep duration at 13 years of age was inversely associated with BMI z-score at 17 years in boys (β=-0.123, 95% CI= -0.233, -0.012) and positively associated with changes in BMI z-score between ages 13 and 17 years in girls (β=0.050, 95% CI: 0.002-0.097)  After adjustment for adiposity at baseline these results were no longer statically significant. | Parental education, KIDMED index and BMI z-score at baseline |
| Roberts and Duong, 2015  USA | The Teen Health 2000 cohort study (N=3134)  Age at baseline: 11-17 years  Follow-up: 1 year | Sleep restriction (≤6 hours of sleep/night) | Interview | Obesity status at follow-up | Sleep restriction did not increase future risk of obesity. | Age, gender, family income, major depression and obesity at baseline. |
| Lytle et al, 2012  USA | Two longitudinal cohort studies:(1) The Identifying Determinants of Eating and Activity and (2) Etiology of Childhood Obesity (N=723)  Mean age at baseline: 14.7 years of age  Follow-up: 2 years | Sleep duration | Sleep: based on 2 questions from the Night Eating questionnaire (validated) | Two-years change in BMI (kg/m^2^) | There were no statistically significant associations between change in total sleep and change in BMI over time. | Energy intake, PA, screen time/ sedentary behavior, depression and socioeconomic status, race, grade, study and pubertal status. |
| Chen and Wang, 2016  USA | The Early Childhood Longitudinal Study, Kindergarten Class (N=7090)  School year at baseline: 5^th^ grade  Follow-up: 3 years | Number of food stores by type of store: supermarkets, limited-service restaurants, small-size grocery and convenience stores. | ZIP-Code Business Patterns data from the Census Bureau in 2004 and 2007 | Change in BMI (kg/m^2^) and obesity status from 5th grade to 8^th^ grade | Girls living in neighborhoods with ≥3 supermarkets had a lower BMI three years later (b=-0.62; 95% CI: -1.05, -0.18; p<0.01) than did those living in neighborhoods without any supermarkets. Girls living in neighborhoods with many limited-service restaurants had a greater BMI (b=1.02, 95% CI: 0.36, 1.68; p<0.01) and odds of being obese (OR=4.45, 95% CI:1.54, 12.83) three years later than did those living in neighborhoods with ≤1 limited-service restaurant. | Baseline BMI/obesity status, baseline age, gender, race/ethnicity, household socioeconomic status, home-moving during follow-up, number of establishments in the ZIP code area, poverty rate, urbanization level, proportion of Hispanic and non-Hispanic Black populations, proportion of foreign-born population, total population size and land area size. |
| Shier and Sturm, 2012  USA | The Early Childhood Longitudinal Study, Kindergarten Class (N=6260)  School year at baseline: 5^th^ grade  Follow-up: 3 years | Three alternative measures od food environment | (1) Counts of a particular type of food outlet per 1000 population (InfoUSA), (2) food environment indices (Retail Food Environment Index and Physical Food Environment Index) and (3) indicators for the presence of specific combinations of types of food stores | BMI percentile in 8th grade and change in BMI percentile from 5th to 8th grade | More types of food outlets in an area were associated with a higher BMI (β=4.05, p<0.05). | Age, gender, race/ethnicity, mother’s education, family income, weekly hours spent watching TV, weekly days of vigorous PA, child-parent interactions and census tract characteristics (median income, percentage non-Hispanic White population and street connectivity index). |
| Macfarlane et al, 2009  Australia | The Health, Eating and Play Study(N=132)  Age at baseline: 10-12 years  Mean Follow-up: 3 years | Family food environment (breakfast eating patterns, food consumption while watching TV, parental provision of energy dense foods and child consumption of energy dense food at home and away from home) | Questionnaire completed by the parents | Three-years change in BMI z-score and obesity status | More frequent dinner consumption while watching TV was associated with a higher BMI z-score (B=0.3, 95% CI = 0.0, 0.6) and less frequent breakfast consumption was associated with higher odds of overweight (OR=2.2, 95% CI=1.1, 4.7). | BMI z-score and obesity status at baseline, gender, maternal education, TV viewing, PA, maternal BMI and clustering by school. |
| Crawford et al, 2010  Australia | The Children Living in Active Neighborhoods Study(N=301)  Age at baseline: 10-12 years  Mean Follow-up: 5 years | Home environment (social support, role modelling, rules and restrictions, physical environment), perceived neighborhood environment (local traffic, road safety, sporting venues, public transport) and features of the neighborhood environment (destinations, road connectivity, traffic exposure) | Features of the neighborhood environment: Geographic Information Systems  Home environment: questionnaire filled by the parents.  Perceived neighborhood environment: Five-point Likert scale filled by the parents. | Five-year changes in BMI z-score | Having non-married parents was associated with higher BMI z-score; In boys, a higher number of sedentary items and time spent in MVPA by mothers was associated with higher BMI z-score; In girls, a higher time spent in SB by mothers was associated with higher BMI z-score, while a higher number of rules regarding SB and number of PA items was associated with lower BMI z-score. | Age at baseline, effects of clustering by school, all factors significantly associated with the outcome in Model 1 and BMI. |
| O’Hara and Haynes-Maslow, 2015  USA | The Early Childhood Longitudinal Study, Kindergarten Class (N=2263)  School year at baseline: 5^th^ grade  Follow-up: 3 years | Vending machine availability | Reported by school administrators | Change in BMI (kg/m^2^) between 5^th^ and 8^th^ grade | Vending machine availability had a positive association with BMI among Hispanic male students (β=0.46, p<0.10) and low-income Hispanic students (β=0.40, p<0.10). | Child has a disability, lives with nonbiological parents, lives with a single mother or father, number of individuals in the household, days per week that the child eats dinner and breakfast with the family, the ratio of income to federal poverty line, lives in a rural region, the percent minority students enrolled in the child’s school, number of hours per week watching TV and number of days per week the child exercised for 20 consecutive minutes. |
| Wardle et al, 2007  United Kingdom | The HABITS study (N=2727)  Age at baseline: 11-12 years  Follow-up: 5 years | Weekly number of school-based physical education sessions | Teacher’s (Year 11 only) and student’s reports (Year 7 and 11) | BMI (kg/m^2^) in Year 11, BMI change (Year 7-11) and overweight/obesity status at Year 11 | There were no significant effects of school physical education on changes in BMI or probability of becoming obese. | Ethnicity, family socio-economic deprivation, age, effect of student’s clustering and baseline values. |
| Assunção et al, 2012  Brazil | The 1993 Pelotas (Brazil) Birth Cohort Study (N=4032)  Age at baseline: 11 years  Follow-up: 4 years | Socioeconomic position (Household Assets index) and weight loss dieting | Interview | Change in obesity status from 11 to 15 years of age | Low-income girls were more likely to become obese from 11 to 15 years of age compared with high-income ones (p_trend_=0.01). For the boys, socioeconomic position was associated with ceasing to be obese (PR=2.7, 95% CI: 1.6, 4.6 for adolescents of the highest tertile). Those who were on diets to lose weight were more likely to become obese (girls: PR=3.3, 95% CI: 1.7, 6.3; boys: PR=4.3, 95% CI: 0.6, 1.9) or to achieve a normal BMI category (girls: PR=5.4, 95% CI: 3.4, 8.6; boys: PR=3.8, 95% CI: 2.5, 5.8) at 15 years of age. | Household Assets index at 11 years of age. |
| Campbell et al, 2010  Australia | The Health, Eating and Play Study(N=188)  Age at baseline: 10-12 years  Mean Follow-up: 3 years | Parental use of feeding restriction | Parental self-report using the Child Feeding Questionnaire | BMI z-score at follow-up | Feeding restriction score at baseline was not associated with follow-up BMI z-score. | Gender, baseline maternal BMI and education and child’s BMI z-score at baseline. |
| Duckworth et al, 2010  USA | Sample from a socioeconomically and diverse public school (N=105)  Mean age at baseline: 10.56 years (5^th^ grade)  Follow-up: 3 school years | Self-control | Self-reported questionnaires | BMI z-score in 8^th^ grade and changes in BMI z-score from 5^th^ to 8^th^ grade. | Self-controlled 5^th^ graders had lower BMI z-scores in 8^th^ grade compared to their more impulsive peers. (β=-0.28, p<0.05) Self-control in 5^th^ grade predicted decreases in BMI z-scores from 5^th^ to 8^th^ grade. (β=-0.15, p<0.05) | Gender, age, socioeconomic status, ethnicity, IQ, happiness and 5^th^ grade BMI z-score (at step 4 only). |
| Cornes et al, 2007  Australia | Participants from a study on melanoma (12 and 14 years) and another on cognition (16 years) (N=1143)  Age at baseline: 12 years  Follow-up: 4 years | Total phenotypic variance decomposed in additive genetics, non-additive genetic, environmental influences shared by members of a family and environmental influences unique to each family member. | Blood samples | BMI (kg/m^2^) at 12, 14 and 16 years | Most of the inBMI phenotypic variances at all 3 ages was explained by genetic factors (additive and/or non-additive) for males and females. | Age, gender and gender by age interaction term. |
| Liem et al, 2010  Netherlands | The TRacking Adolescents’ Individual Lives Survey study (N=1275)  Mean age at baseline: 11.1 years  Follow-up: 5 years | Candidate single nucleotide polymorphisms near the *INSIG2*, in the *FTO* and near the *MC4R* genes | DNA extracted from buffy coats or buccal swabs | Repeated measure analysis of 3 measures of BMI z-score (11 years, 13.5 and 16years) | Common variation near the MC4R (B:0.12, 95% CI=0.03,0.20) and the FTO (B:0.09, 95% CI=0.01,0.16) was associated with BMI in repeated measure analysis. | Age, PA and gender. |
